# Designing a Healthy Food-Store Intervention; A Co-Creative Process Between Interventionists and Supermarket Actors

**DOI:** 10.34172/ijhpm.2021.110

**Published:** 2021-09-11

**Authors:** Cédric N.H. Middel, Tjerk Jan Schuitmaker-Warnaar, Joreintje D. Mackenbach, Jacqueline E.W. Broerse

**Affiliations:** ^1^Athena Institute, Faculty of Science, Vrije Universiteit Amsterdam, Amsterdam, The Netherlands.; ^2^Department of Epidemiology and Data Science, Amsterdam University Medical Centers, Vrije Universiteit Amsterdam, Amsterdam, The Netherlands.; ^3^Upstream Team, Amsterdam University Medical Centers, Amsterdam, The Netherlands.

**Keywords:** Co-Creation, Co-Design, Healthy Food-Store Intervention, Environmental Intervention, Dietary Behaviour, Netherlands

## Abstract

**Background:** Without consideration for the food system in which healthy food-store interventions (HFIs) are implemented, their effects are likely to be unsustainable. Co-creation of HFIs by interventionists and food-store actors may improve contextual fit and therefore the effectiveness and sustainability of interventions, but there are few case studies on the topic. This study aims to provide insights into the integration of knowledge from contextual actors into HFI designs, through a co-creative process, to illustrate potential challenges, advantages, and outcomes.

**Methods:** We describe the co-creative design of an HFI in a Dutch supermarket chain, conducted through three increasingly in-depth design phases. Each phase consisted of a cycle of theorizing (gather insights from literature, feedback, and pilot studies), building (develop intervention designs), and evaluating (interviews or workshops with supermarket actors, to explore barriers and facilitators for sustainable implementation), feeding back into the next phase (drafting adapted intervention designs, based on feedback, and research input). Interview transcripts underwent a qualitative content analysis.

**Results:** We co-creatively designed four types of interventions to promote healthier food choices in supermarkets: (1) price strategies, (2) product presentation and positioning, (3) signage, and (4) interactive messaging. Interventions were aligned with the culture, structures and practices of the supermarket chain, while simultaneously challenging these system characteristics. For example, the idea of price promotions on healthy foods was well-received and encountered only practical barriers, which were easily resolved. However, the specification of tax-like price increases on unhealthy foods led to substantial resistance on cultural and commercial grounds, which were resolved through support from a key supermarket actor.

**Conclusion:** Our results illustrate the potential benefits of co-creation approaches in HFI design. We reflect on the value of more easily accepted interventions to develop collaborative momentum and more radical interventions to drive more substantial changes.

## Introduction

**Key Messages**

** Implications for policy makers**

- Current design and implementation methods for healthy food-store interventions (HFIs) often do not take into account the wider system in which they are implemented.
- This may limit long-term effectiveness, sustainability, and possibilities for upscaling of these interventions.
- Co-creation, by interventionists and food-store actors, provides the opportunity to align the interventions with the wider food-store system, thus facilitating longer-term sustainability and up-scalability.
- In this case study we explore barriers and facilitators for implementation, and potential responses, through co-creation.
- Co-creative design has the potential to realize the acceptance of more radical intervention experiments by food-store actors, through a combination of building momentum and pushing boundaries.

** Implications for the public**

Healthy food-store interventions (HFIs) can help consumers make healthier food choices. However, food-store organisations are part of a wider food-store system whose primary focus is not health promotion, potentially causing unforeseen implementation barriers for the intervention. Interventions designed solely by health-promotion interventionists, who have incomplete knowledge of these potential barriers, may therefore suffer from limited and short-term effectiveness. Co-creatively designing these interventions with actors from that context (eg, supermarkets) may facilitate embedding of the intervention in the food-store system, thus enhancing sustainability and impact. Our findings and reflections may help future interventionists in designing interventions which can more effectively contribute to healthy dietary behaviour and public health.

Healthy food-store interventions (HFIs) have the potential to promote healthier diets,^1^ which contributes diet-related chronic-diseases prevention.^2^ Such interventions are likely more effective and have a broader reach, compared to individual-focussed efforts, due to the widespread promotion of unhealthy foods (over)consumption in the modern food-environment.^3,4^ Unfortunately, HFI implementation faces substantial challenges.

 HFI studies report various context-related implementation barriers (eg, commercial values, incompatibility with existing practices, limiting physical structures, insufficient resources) and relatively few (actively leveraged) facilitators (eg, health-promotion values, relevant expertise, flexible practices).^5^ We argue that these issues arise from an insufficient understanding of the systemic context in which these HFIs are implemented, leading to low alignment between intervention and context, which undermines effectiveness, sustainability and scalability.^6,7^ The systemic context of HFIs is ‘food-store systems,’ which we define as: networks of actors and interactions involved in providing food to consumers, through retail, at a certain scale (eg, local, national, global).

 To understand the implementation problem, we conceptualise food-store systems as constellations,^8^ which can contain smaller constellations (eg, suppliers, stores, food-authorities), and be part of larger ones (eg, broader food-systems).^8^ Constellations are characterised by internal *cultural* (values, beliefs) and *structural* (rules, boundaries, resources) elements, with varying levels of strength and importance.^8^ These ‘*structuring elements*’ guide the activities of *actors* (people) operating within the constellation, and the tangible actions (*practices*) they produce.^8^ Practices also reproduce the structuring elements which created them, leading to a self-reinforcing cycle, making constellations resilient to change. Furthermore, constellations have interactions *external* to their boundaries, eg, other constellations, or large environmental events, which can pressure structuring elements to change or develop.^8^

 When HFIs are implemented in a food-store constellation, dissonance between the intervention and structuring elements of the constellation becomes implementation barriers.^5^ Because structuring elements are often implicit,^8^ such cases of dissonance are difficult to anticipate for outside interventionists (HFI-design, implementation, and impact researchers). This leads to barriers during implementation, which could have been resolved in the design-stage. Recent literature proposes stronger involvement of food-store actors in HFI-design,^9,10^ to leverage their implicit knowledge of the food-store constellation. In this study we explore the operationalization of this idea through co-creation.

 Co-creation is an approach often used in intervention design.^11^ Its aim is to combine knowledge through collaborative social practices (eg, problem-solving) between academic (eg, interventionists) and non-academic stakeholders (eg, food-store actors).^12,13^ The underlying assumption is that social interaction drives the integration of the stakeholders’ (implicit) knowledge, and stimulates ownership over the end-result.^12,13^ The end-result (eg, an HFI design) is thus understood and supported by its stakeholders, and fits its intended context (eg, food-stores). Co-creation has shown positive influences on intervention impact, outcomes, and sustainability in healthcare and services.^14-17^ Furthermore, it is applied in HFI research,^18^ to address evidence gaps and develop new knowledge.^19^

 In summary, co-creative HFI-design could facilitate an intervention to fit into its implementation context more successfully, benefitting impact, outcomes and long-term sustainability.^14-17,19^ Co-creation approaches for HFI design are underdeveloped in the literature,^15^ with little in-depth information on operationalisation and outcomes, indicating a knowledge gap. Our study aims to reduce this gap through addressing the following question: “How does identifying and addressing sustainable-implementation barriers and facilitators through the use of a co-creation approach impact the process and outcomes of designing an HFI?” This question will be answered through the case study of a co-creative HFI-design process.

## Methods

 We followed the Standards for Reporting Qualitative Research.^20^ The following sections describe: the study context, approach and design, analytical framework, data collection (materials, participants, methods), researcher characteristics, ethics, data processing, data analysis, and internal validity.

###  Context

 This study was part of the SUPREME NUDGE project,^21^ which aims to improve cardiometabolic health among Dutch low-socioeconomic-status adults by developing a sustainable HFI.^21^ The project is a collaboration of Dutch universities, medical centres, and health organisations, with researchers from epidemiology, nutrition, systems science, and psychology.

 In the Netherlands, supermarkets are the primary food source for consumers, with over twenty chains, and market shares ranging from 34.7% to >0.5%.^22^ Supermarket density, and competition over customers, is high. Marketing is price-focussed, reinforced by ‘price comparisons’ between chains in Dutch media. Due to repeated ‘price wars,’ profit margins for most Dutch chains are low, and employee workload high.^24^ Recently, promotions around health (eg, discounting fruits and vegetables) have become more prevalent.^25,26^

 SUPREME NUDGE collaborates with supermarket chain Coop, a middle-sized chain (4% market share in 2019),^27^ mostly located in rural areas. Half their stores are independent franchisers.^28^ The organisation is a ‘cooperative’ (no shareholders), meaning profits are reinvested in the organisation and benefits for its customers,^29^ and therefore often works on societal issues (eg, health promotion) put forward by their customers.^28^

###  Approach and Design 

 As explained in the introduction, we followed a co-creation approach for HFI-design. The academic stakeholders were project-interventionists, including the authors. The non-academic stakeholders were actors in the Coop organisation (‘supermarket actors’). The collaborative social practice was designing a sustainable and effective HFI, with the underlying aim to gain integrated knowledge on structuring elements which can pose barriers/facilitators towards HFI sustainability.

**Figure 1:**
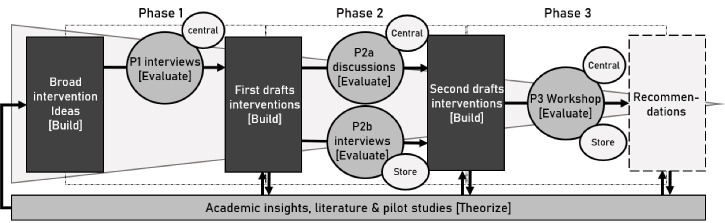
A Visualisation of the Study Approach, Illustrating the Three Phases, and Their Internal Theorizing, Building, Evaluating Cycles. Detailed information on the phases can be found in Table 1.

 The design process was organized in three phases (Figure 1), organised as increasingly-specific iterations of the Design Research Cycle:(21) Phases started by interventionists developing insights from literature and pilot studies (‘theorize’(21)). Based on insights (and previous feedback), interventionists developed or adapted HFI designs (‘build’(21)). These designs were then evaluated through discussion with food-store actors (‘evaluate’(21)), providing feedback for the next phase.

 Phase 1 (P1) explored the Coop constellation, and explored broad ideas for HFI designs with central-management actors, exploring their views on customer reactions, effectiveness, and discussing the operationalization of these ideas in their organisation. Phase 2 (P2) explored concerns and conditions related to the implementation and effectiveness of HFI designs, and how to address these in the design, from a central-management (P2a) and store-management (P2b) perspective. Phase 3 (P3) comparatively evaluated the developed designs and solutions, and discussed the relative feasibility and effectiveness of designs, to recommend which designs should be developed and piloted further, in real stores. Table 1 summarizes each phase.

**Table 1:** Overview of the Study Phases and Their Characteristics^a^

**Phase**	1	2a	2b	3
**Date**	May 2018-September 2019	March-May 2019	April-May 2019 February 2020 (follow-up)	June 2019
**Goal**	Exploration of systemic context. Discussion of broad intervention ideas and how these fit into the systemic context	Discussion of specific interventions, concerns and conditions for their implementation and effectiveness, and how to address these in the designs	Final comparative evaluation of interventions, through scoring perceived feasibility, and outcomes. Collect recommendations for adjustments and further development
**Participants ** *(background)*	10^b^ (formula management, marketing, corporate social responsibility, store operations, and product management)	12^b^ [4 returning from P1] (board members, division management, formula management marketing, corporate social responsibility, store operations, and product management) 2^d^ (Interventionists with expertise on discussed designs)	17^c^ (store management and owners)	10^b^[3 returning from P1/2a] (supply management, business analytics, space management, regional management, formula management, marketing, corporate social responsibility, store operations, and product management) 2^c^[2 returning from P2b] (store management and owners) 3^d^ (Interventionists with expertise on discussed designs)
**Data Collection Method ** *(frequency; avg. length)*	1- participant interviews (4; 1h); 2- participant interviews (3; 1h)	Leadership meetings (2; 1h); Group discussions (4; 1h)	1-participant interviews (17; 1h)	Workshop (1; 2h)
**Data Collection Materials ** *(function)* (See Supplementary file 1)	IGp1 (interview guide); Broad ideas (discussion piece)	IGp2a (interview guide); Prototype Guide (discussion piece)	IGp2b (interview guide); Summary (discussion piece)	IGp3 (interview guide); Extended summary (discussion piece); Scoring matrix (tool)

^a^ For more information, refer to the appropriate section; ^b^Participants at central-management positions; ^c^Participants at store-management positions; ^d^ Academic interventionists working at the SUPREME NUDGE project.

###  Analytical Framework

 We used a co-creation approach to identify structuring elements which could pose barriers or facilitators to HFI sustainability. The Constellation Perspective,^8^ described in the introduction, was used to describe HFI implementation.

**Figure 2:**
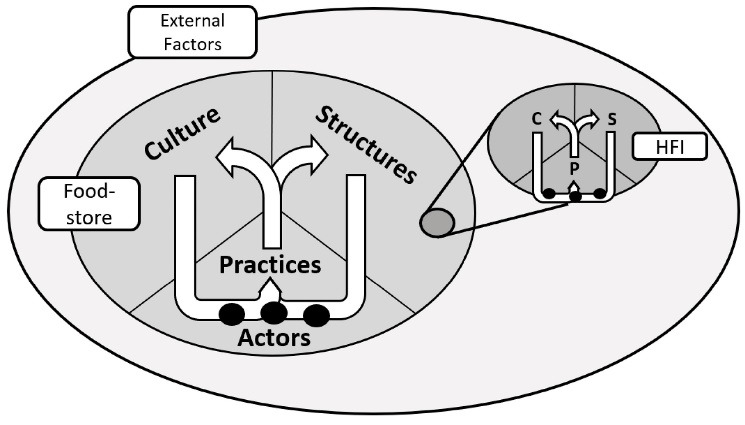
HFI Implementation Conceptualised Through the Constellation Perspective. Shown are external influences (outer circle), and the two constellations of a food-store organisation (left inner circle) and an HFI localised inside the food-store organisation (right inner circle). Abbreviation: HFI, Healthy food-store intervention.

 We conceptualise HFIs and food-store organisations as constellations within the food-store system. HFI implementation represents merging these constellations. The structuring elements and practices of both constellations can align (being similar, or supportive) or misalign (being opposed somehow), and thus become, respectively, facilitators or barriers to sustainable implementation. Depending on the relative strength and importance of involved elements, barriers/facilitators can be superficial or fundamental in the constellations, requiring appropriate levels of concession and adjustment in the HFI design to address. Figure 2 illustrates this conceptualisation. In this paper we focus on the elements of the food-store constellation. This framework enables us to structure the interacting actors, factors, and mechanisms involved in HFI implementation.

###  Data Collection

 We discuss participant recruitment and data collection methods, per phase. Our methods drew inspiration from reflexive monitoring,^30^ human-centred design,^31^ and co-design,^32^ which similarly engage stakeholders’ inherent knowledge. We sought to develop insight through collaborative HFI-design between interventionists and supermarket actors: a co-creative process. Table 1 summarizes dates, times, participants, materials, and other details, Table 2 summarizes interventions. Interview guides, and descriptions of discussion pieces and tools can be found in Supplementary file 1. The primary data collected was audio-recordings of the interviews and discussions with and between participants.

**Table 2:** Summary of the Interventions Featured in the Discussion Pieces in Each Design Phase

**Phase 1**	**Phase 2a**	**Phase 2b**	**Phase 3**
Price strategies Price increases and decreases to deter unhealthy and stimulate healthy choices.	Subsidy: The decrease of prices to stimulate certain choices.		
	Tax: The increase of prices to deter certain choices.	Tax: The increase of prices to deter certain choices, always combined with a subsidy on a healthier alternative.	
Presentation & positioning Adjustments in product positions, availability, or presentation on shelfs, or presentation of healthy products in alternative places through the store.	Shelf positioning: Adjustments in (relative) shelf position, or visible number of products to influence choices.		
	Second placement (baskets/end-of-aisle): Presentation of healthy products in the store, in addition to their regular shelf position, eg, in baskets near the check-out, or end-of-aisle shelfs.		
	Meal suggestions: Placement of healthy alternative products in existing meal suggestion presentations.		
	Healthy check-outs: Replacement of the ‘impulse’ products offered at check-out by healthier options or non-food products.
	Scarcity nudge: Placing a limit on number of a product customers can buy, or otherwise limiting the amount of a product seemingly available, to create a sense of value.	*Design discarded*	
	Default nudge: Making unhealthy products more difficult to reach compared to healthy options, eg, through placement behind a counter, or requiring customers to ask for them.		*Design discarded*
Signage & communication The use of signage throughout the store or near products to draw attention, communicate a theme, or provide general or specific information or feedback.	Symbols (health): Symbols which indicate certain characteristics of a product, related to their healthiness. Symbols are explained through posters, flyers, and other signage, and presented in combination with a specific product or group of products.		Symbols (non-health): Symbols which indicate certain characteristics of a product, related to non-health aspects such as taste or ease of preparation. Symbols are explained through posters, flyers, and other signage, and presented in combination with a specific product or group of products.
	Shelf signage (tags, banners, cards, arrows, sticker under product): Various types of signage, meant to draw attention to a part of the shelf, or towards a specific (group) of products, or provide feedback.		
	Door signage (frames): Signage on the glass doors of cooled and frozen sections. Meant to draw attention to (groups of) products.		
	Large signage: Tabs between vegetable and fruit crates, pictures on gates, posters, flyers. Can depict healthy products as inspiration, portion size examples, present promotions, or cooking instructions/information.		
	Signage in/on carts/baskets: Signage in (eg, placemats) or on the sides of shoppingcarts/baskets, depicting healthy products, meals. Alternatively, signage at the carthandles can present a legend for the symbols intervention.		
	Floor/ceiling signage: Stickers/projections on the floor, or tabs on the ceiling, promote certain products, or indicate areas with healthy products.		*Design discarded*
Interactive The use of multi-media techniques seeking to draw attention, provide feedback on choices, or familiarise products, which depend on customer input or interaction.	Tastings:Presentations where customers can taste certain products, and possibly see how to prepare it, possibly organised by experienced marketing bureau.		
	‘Gaze-nudge’ An affordance nudge in the form of an animated character on a monitor inviting people to choose healthier products.		
	Feedback (receit) Customers receive feedback on product choices by means of a message on their check-out receit.		*Design discarded*
	Dynamic crates The use of a mechanism to make vegetable crates tilt upwards in response to approaching customers.		*Design discarded*
	*Not yet conceived*	Providing shopping list materials: Paper and pen are provided at the start of the store, to facilitate the use of shopping lists, for more deliberate choices.	*Design discarded*

Legend:Underscored: intervention was discarded or substantially adjusted. *Italics*: component was included in final recommendations

####  Phase 1 (Participants)

 We purposefully sampled ten participants from backgrounds in supermarket-organisation central-management with relevance to intervention ideas. Participants were interviewed alone, or in pairs (in case of similar/connected background). Sampling was concluded when data saturation was reached (no new insights in 2 subsequent interviews) and all relevant perspectives were represented. We focussed on central management as these actors have the broadest view of the organisation, and are the primary decision-makers.

####  Phase 1 (Methods)

 We conducted one-hour interviews with the participants in their capacities as potential intervention-users, and organisational-context experts,^32^ following a system analysis methodology.^30^ Interviews first explored the general context of the participant and the supermarket-organisation, by discussing their work and important connections. Next, participants were asked to share their views on health promotion in the Dutch supermarket environment, with follow-up questions exploring problem definitions, barriers, facilitators, causes, and solutions. These questions identified constellation and external elements with relevance to the (un)healthy environment problem.

 Following the ‘Conversation Starters’ method,^31^ participants were introduced to several broad ideas for health promoting interventions, and asked to reflect on the perceived feasibility, acceptability, and effectiveness of these ideas. Follow-up questions explored the underlying reasons. This line of questioning linked the identified constellation elements to the intervention ideas.

####  Phase 2a (Participants)

 We engaged twelve key decision-makers in supermarket-organisation central-management, in groups of 3-5 participants. Participants were proposed by our contact-person as the appropriate decision-makers. No new participants were sought later in the process. Instead, repeated discussions with the same participants were held until agreements on various intervention design aspects were reached. Two interventionists (alongside the authors) from the SUPREME NUDGE project participated as intervention experts.

####  Phase 2a (Methods)

 We conducted one-hour group-discussions with the participants, in their capacity as potential intervention-users, organisational-context experts, and key decision-makers.^32^ The first two discussions were part of organisational leadership meetings, where official approval to continue the development of specific interventions would be determined. Due to our status as ‘guests’ in these meetings, we did not follow an interview guide. Following a ‘Gut Check’ method,^31^ a summary of interventions was presented to the participants, the feasibility and potential risks of which were discussed, with the researchers primarily answering questions on intervention designs, but at times interjecting the discussion with deepening questions. Finally, a selection was made of interventions to develop further, and certain boundaries. The discussion and selection provided insight into which structuring elements were considered vital, and which more flexible, when in conflict with intervention designs.

 The remaining four group-discussions were held with key decision-makers and one interventionist (alongside the first author), combining ‘Rapid Prototyping’ and ‘Integrate Feedback and Iterate’^31^ elements to evaluate and develop interventions. Two group-discussions focussed on ‘price’ interventions, and two on ‘nudging’ interventions. Group-discussions used prototype guides for interventions (based on P1 feedback, pilots, and literature) as discussion pieces, which covered intervention designs and targeted products and were shared in advance. The relevant interventions (nudging or price) were evaluated, examining concerns with the described plans and designs, in depth. Participants discussed the origins of concerns, and intervention-design solutions. Between group-discussions, received feedback was incorporated in the guides. Through this method, aspects of the supermarket-constellation which misaligned with (characteristics of) interventions were identified, explored, and solutions discussed.

####  Phase 2b (Participants)

 We sampled seventeen managers and private owners of affiliated stores. A list of 30 approved stores was provided by our contact-person, based on the owner/manager’s perceived suitability, and client-base characteristics (low socioeconomic status). Participants were recruited until data saturation was reached (no new insights in 2 subsequent interviews). Seven people declined participation due to being occupied or uninterested. A follow-up was held with managers of two new potential pilot stores, which yielded no new insights.

####  Phase 2b (Methods)

 We conducted one-hour interviews. We combined ‘Gut Check,’ and ‘Integrate Feedback and Iterate’ elements^31^ to explore the sustainability of interventions in the store environment. Interviews were conducted at participants’ stores, to gain a sense of the intervention environment.^32^

 Participants were presented with a ‘summary’ of interventions, as a discussion piece. Participant were asked to share their thoughts on these interventions. We explored the feasibility of each intervention in participants’ stores, and their perceived health-promotion effectiveness. Through deepening questions, the researcher explored the underlying constellation elements. When an intervention was perceived unfeasible or ineffective, we would explore the reason, and possible intervention-design solutions. Finally, participants were asked if they had additional suggestions for interventions. Between interviews, adaptions were made to the summary discussion-piece, removing interventions with strongly negative feedback, and adding new ideas.

####  Phase 3 (Participants)

 We purposefully sampled twelve participants from the supermarket-organisation departments involved in the (hypothetical) implementation of the discussed interventions. A list of departments was drafted in collaboration with our organisational contact, who then recruited representatives for each. Three interventionists (alongside the authors) from SUPREME NUDGE participated as intervention experts, and group leaders.

####  Phase 3 (Methods)

 We held one workshop, facilitated by the first author, which combined elements of ‘Gut Check’ and ‘Integrate Feedback and Iterate’ methods^31^ with a ‘scoring matrix,’ to comparatively evaluate and prioritize interventions, from the perspective of implementation-context experts and potential users.^32^

 Participants were divided into three groups (selected in advance), of participants from connected departments. Each group included one interventionist, and received an ‘extended summary’ discussion piece, detailing interventions relevant to the group’s represented departments. The primary data collected in this phase was recordings of the discussions between participants. Each group performed the same exercises, for their assigned interventions: First, they drew a ‘scoring matrix’ tool, with two axes, ranging from ‘positive,’ to ‘negative,’ representing perceived implementation feasibility, and combined value of outcomes (health and commercial). The sum of these scores was regarded as the ‘sustainability’ of the intervention. Groups would assign (relative) positions in the matrix to their assigned interventions, by writing them on post-its, and placing these in the matrix. When an intervention scored negative on an axis, the group would discuss what could be improved in its design to achieve a positive score on the axis, and add a post-it, detailing the intervention and this condition, on the matrix, in the spot where this ‘improved’ intervention would belong. For interventions that scored positive on both axes, suggestions for improvement could also be added if desired.

 After all interventions were discussed, groups made a selection of interventions they felt should be prioritized for further development efforts, and which (if any) improvements should be made to make these interventions more sustainable. In a plenary session, the groups presented their matrixes to each other, and explained their choices, suggestions, and final selection. Other groups could provide feedback on these choices. Outcomes of this plenary discussion were summarized on a new scoring matrix with post-its, to visualise the final recommendations of the participants.

###  Researcher Characteristics

 Several researcher characteristics could have influenced the data collection and analysis: Data was primarily collected and analysed by the first author, a PhD student in systems innovation and transition theory, with training and experience in qualitative methods and previous experience working in a supermarket environment. Furthermore, the research activities were formally supported by the Coop leadership. These characteristics arguably facilitated data collection rigour, and the openness of participants.

 The third author conducted several interviews in P1 and P2b. She is a senior researcher who initiated the collaboration with the supermarket-organisation. Her data collection rigour was likely facilitated by her training and previous experience in conducting qualitative studies.

 The three interventionists participating in P3 had backgrounds in epidemiology (one full professor, two PhD students). They had previously been involved in the theorizing on which the interventions were based. The discussion depth in their groups arguably benefited from this background. Although their involvement in the designs may have been a deterrent for criticism, participants did not seem hesitant in providing negative feedback.

###  Data Processing

 Interviews, group-discussions, and workshop were audio-recorded and saved on a secure server. Recordings were transcribed word-for-word, and the transcripts were saved on the same server. Finally, anonymized versions were made of each transcript by removing all identifying information.

###  Data Analysis

 The anonymized transcripts were analysed in Atlas.ti coding software.^33^ The unit of analysis was barriers and facilitators for HFI sustainability and their constellation-element origins. We identified these through a qualitative content analysis of interviews, group-discussions, and workshop transcripts.^34^ We followed an inductive approach, recommended when knowledge on the studied phenomenon is lacking or fragmented.^34^ Our predefined theoretical framework is a deductive element,^34^ but not leading in the analysis, rather meant to relate findings back to the broader theoretical rationale of the study.

 Preceding analysis, codes were developed for the major concepts of our analytical framework (*culture, structure, practice, actor, external, barrier, facilitator*) and the interventions (Table 2). First, transcripts were open-coded by the first author, to describes themes in barriers/facilitators (including the interventions they applied to), and related constellation elements. Co-occurrences between codes were noted as potential interrelations between barrier/facilitator and constellation themes. At frequent (~5 transcripts) intervals, codes and noted interrelations were critically reviewed, and codes for subthemes and broader themes were added where appropriate. Codes were marked for the study phase(es) where they occurred, to allow chronological stratification.

 For synthesis, we categorized constellation codes under their respective constellation elements, creating a hierarchical tree.^34^ We placed the barrier/facilitator codes in the tree, and operationalised the previously noted interrelations between codes as additional connections in the tree. These connections indicated which barriers/facilitators emerged from which constellation elements, and how constellation themes related between themselves. Based on the tree, we drafted two summarizing tables of our findings.

###  Internal Validity

 Three measures were taken to guard internal validity: We made field notes of all interviews, in case recording failed. Furthermore, we performed ‘member checks’ for the interviews by drafting summaries of the points of interest and asking the interviewee to evaluate our interpretation. No substantial revisions were requested. Finally, transcript coding was triangulated between the first and second author: after coding half of the transcripts, the coding critically discussed with the second author regarding described themes and connections.

## Results

 We first discuss the major themes which emerged in our discussions to identify and address sustainability barriers and facilitators for the HFI designs. We discuss themes separately for each intervention group (see Table 2). Subsequently, we explore themes which were observed more broadly, and likely more ‘systemic’ in nature. Supplementary file 2 summarizes all barriers and facilitators and the related interventions. Supplementary file 3 summarizes barriers and facilitators categorized according to underlying constellation elements.

###  Price Strategies

 This section focusses on price strategy interventions, which are ‘price increases (subsidies) and decreases (taxes), which deter unhealthy and stimulate healthy choices.’ The major themes of discussion were cultural acceptance, product profitability, and price-management system integration.

####  Cultural Acceptance

 Subsidies were readily accepted as a potential intervention, due to their perceived effectiveness, and similarity to existing discount practices. In contrast, early on (P1), taxes were regarded as a “taboo,” and generally disliked by participants. The reasoning was that taxes could feel punitive and judgemental towards customers, driving them away. An additional fear was that the regularly published ‘price comparisons’ between supermarket chains by Dutch media, often including potentially taxable unhealthy products, would negatively impact customer satisfaction. These arguments were repeated by the store-level participants in P2b.

 “*There are price-measurements, consumer guides, there are websites where prices are compared. It’s about tenths of a percentages, but costumers are very sensitive to this. Customers have no idea how much things cost; price is perception” *[A8; central].

 Due to the strong evidence for the effectiveness of taxes, the interventionists elected to keep looking for solutions, in spite of cultural resistance. In this process (P2), two facilitative developments occurred: (1) We contacted a major consumer organisation involved in the price comparisons, and found them willing to exclude intervention stores from their comparisons during initial pilot stages; (2) A major organisational leader expressed their support for piloting taxes, regarding them as a minor risk, given the limited number of pilot stores, and potential knowledge gain (P2a). This support stimulated openness among previous opponents, to explore the idea.

 “*Maybe, in that case, I should be a bit less rigid [regarding price increases]” *[A10; central].

*“No, yes, [major org. leader] didn’t mind as much…He even said ‘why not more stores?’” *[A2; central].

 The now open-up discussion allowed us to find a compromise (P2a): taxes would be implemented next to a subsidy on a similar but healthier product. This was regarded as more customer friendly, by offering a similar product instead of simply punishing certain preferences. Furthermore, it would keep sales within a product group (preventing negative effects on product-group managers’ performance metrics), and the direct contrast between taxed and subsidised products could increase impact. This design received further support throughout the process (P2-3).

####  Product Profitability

 Another theme was product profitability. Initially (P1-2), participants had concerns regarding the impact of subsidies on the profit margins of subsidized product. Some believed that smaller margins would be compensated by increased sales volume, but others contested this. When, in P2a, the pairing of taxes and subsidies was proposed, this was also regarded as a solution to balance the costs of subsidies with increased profits from taxes, especially because taxed products were perceived as generally in higher demand.

 “*I would find the proposal more acceptable if it would indeed have the healthy face the unhealthy. Because then it becomes visible that the [price] increase you get for one is compensated with the other*” [A18; central].

 Regardless, central-management participants desired close monitoring of the financial impact of the intervention, to possibly intervene if losses were too large, demonstrating that some concerns remained.

 In the same discussions (P2a), the size and frequency of taxes and subsidies came up. Building on the idea of pairing taxes and subsidies, a maximal price difference (25%) between healthy and unhealthy alternatives was agreed upon, through subsidies and taxes or solely subsidies. The agreed limits lowered concerns regarding profit margins (for subsidies), and potential negative customer feedback (for taxes). Although differences in product and sales distribution product required group specific plans to be developed on a case-by-case basis, these general agreements were a major step forward in the discussions.

####  Price-Management System Integration

 A final theme was the integration of pricing interventions in the price-management system. In P1-2 we learned that store prices are centrally regulated, and that local deviations (particularly taxes) are difficult.

 “*Usually, we say: ‘pricing up, we don’t do that.’ That’s not even possible in our system. So, for that you need the headquarter” *[A14; central].

 In P2a, we found that the organisation was planning a switch to a new system, which likely could maintain local price-increases and decreases. However, in P3, and the coronavirus disease 2019 (COVID-19) pandemic thereafter, the implementation of this system was delayed multiple times, making the selection of pilot stores for the price strategies difficult.

###  Presentation and Positioning

 This focusses on presentation & positioning interventions, which are ‘adjustments in product positions, availability, or presentation on shelfs, or presentation of healthy products in alternative places through the store.’ The major themes of discussion were planogram system integration, space constraints, and product characteristics.

####  Shelf-organising System Integration

 A central theme was the dictation of product shelf-positions. These positions are dictated by centrally developed and maintained planograms, which the stores implement. These plans also inform the automated stock-replenishment system in its predictions of which and how many products to order. Deviations are possible, but if not organisation-wide they require individual stores to manually correct replenishment orders, causing substantial extra work. This system presents a barrier for initial intervention pilots conducted in a selection of stores, noted frequently in P1-2, but is also a tool for long-term sustainable integration. Central-management (P1) and store-management (P2b) participants indicated the intervention should be integrated in this system from the start, for consistency and limiting workload for stores.

 “*If you want [to change shelf positions], you would need to do it that way [through planograms], because then you integrate it in our current processes. Then you can track sales, our automatic supply system can take it into account” *[A8; central].

 Unfortunately, central-management participants (P2a) explained that planograms take substantial time to maintain, and the department was loaded near maximum capacity. This was problematic, as the intervention stores would require new planograms for every adjusted shelf. The problem was compounded by the fact that not all stores use the same planograms, due to shelf-length differences. For our initial pilot, it was agreed upon that intervention planograms for three shelves could be altered. For long-term sustainability this was not an issue, as, in case of organisation-wide implementation, the existing planograms could simply be adjusted.

 “*We need to maintain [the planograms] here [centrally], because, as you said, our supply-chain and stocking is linked to them. So that has an enormous impact. Maybe we should make a selection, if we could make one or two, dedicated for you” *[A11; central].

 Another consideration was that planograms balance multiple metrics, including restocking efficiency, profits optimization, and supplier input. Participants (P1-2) emphasized these needed to be taken into account in the intervention planograms, to avoid additional workload for stores, financial losses, or conflict with suppliers.

####  Space Constraints

 Another major theme was space constraints, which impacted ‘second-placement’ interventions, eg, presenting healthy products in baskets, end-of-aisle shelves, or at check-outs. Although well-proven concepts, there were multiple barriers: Store-management participants (P2b) noted that floor space in stores is highly contested, which constrains the number of these interventions: *“Yes, and where are you going to put it?” [B8; store]* Furthermore, participants (P2-3) explained that baskets and end-of-aisle are usually reserved for specific products (season, own brand, promotions). A facilitator was that these products often include healthier options, which could be focussed upon. It was agreed to reserve 20%-30% percent of baskets, one end-of-aisle shelf, and 50% of check-outs, in each store, for interventions, using the healthier options in the usual product range.

####  Product Characteristics

 Another recurring theme was the characteristics of products interventions focussed on. Participants (P2-3) noted that some products have temperature requirements, short shelf-life, or an uncommon shape, which makes certain presentation & positioning interventions more difficult. Some solutions were proposed, such as movable coolers, but remained an additional hurdle. Furthermore, participants knew from experience that, certain products perform better or worse in certain spots, based on price and dietary function. These factors needed to be taken into account in the selection of products and spots for interventions.

 “*But non-food is essentially a no-go, because it doesn’t work. It’s on the shopping list, it’s deliberate. The check-out is no place for them” *[B1; store].

 Finally, participants (P2-3) regarded some healthier products as lower in demand, and therefore not as financially attractive when used in an intervention. Throughout the design process, participants indicated how their organisation lacked the knowledge to accurately select healthy products. As a solution, product selection would be informed by the interventionists, but this could present a barrier for future upscaling. As solution, the involved parties agreed to collaborate with nutrition authorities.

###  Signage

 This section focusses on signage interventions, which are ‘the use of signage throughout the store or near products to draw attention, communicate a theme, or provide general or specific information or feedback.’ The major themes of discussion were guidelines, health authority, customer preferences & beliefs, and workload and maintenance.

####  Guidelines

 The organisational ideas and beliefs on how stores should be experienced by customers, including signage communication, are institutionalised in a set of guidelines: the ‘formula.’ These guidelines constrain signage-intervention design options, but also presents a facilitator, as they carry legitimacy within the organisation, and represents valuable experiential knowledge. Throughout the design process, participants indicated that interventions should fit into the formula, as they endorsed and trusted its guidance on ‘effective’ signage. Fortunately, most signage interventions fitted the guidelines with minor adjustments. Notably, the guidelines had recently been revised, and the old style was often cited as ‘what not to do:’

 “*So what [ organisation ] did is they removed a whole lot of signage, with one idea in mind: ‘more is not always better’” *[B1; store].

####  Authority

 Another theme was the content of signage. Through P1-2b, central and store-management participants noted several problems. Health-related signage was perceived as problematic, because participants felt their organisation lacks expertise and authority on the subject, and believed consumers would distrust such statements from a supermarket chain. Furthermore, if certain claims were be proven wrong in the future, or signage was placed at the wrong products, this could damage their public image. Participants proposed that legitimisation of such information by a recognised authority could resolve this barrier.

 “*If you read [health information] in a retailer magazine, with a picture of someone who tells something, as a baker, with their picture next to it, people think that’s an actor, or they just look good. You [food-store] lack that bit of authority” *[A6; central].

 An accompanying limitation was that health claims regarding specific products are bound by legal restrictions. As a solution to these combined barriers, it was collectively decided that product-specific signage would avoid health-related statements, instead focussing on taste, popularity, or convenience.

####  Customer Preferences and Beliefs 

 During P1, pilot studies indicated that, although customers say they value healthiness, other characteristics (eg, taste, price) often seem to be more influential in their dietary choices, confirmed by various store-management participants (P2b). This was an additional motivation for the decision to refocus product-specific signage on non-health characteristics, as discussed above.

 “*For example, a large part of Dutch customers ask for organic, but when they get to the store and see how that product is many times more expensive than the regular one, they abandon those ideals pretty quickly” *[B5; store].

 There was also discussion regarding more general signage. Participants in all phases expected signage to facilitate healthy choices to be appreciated by customers. Therefore, the signage interventions could be beneficial by improving public image and drawing more customers. However, among store-management level participants (P2b) there were concerns about discussions and conflict with customers over the content of signage. *“The last thing I want is for customers to get into discussion with my employees about what is or isn’t healthy” *[B1; store]. Based on these points, it was agreed to move away from health-focussed signage in general.

####  Workload and Maintenance

 Workload was a recurring theme. Throughout the design process, participants noted how product specific signage (eg, shelf tags) can be labour-intensive to implement and maintain. *“Very fun, but don’t get the time to work on that” *[B12; store].Store-management participants (P2b) explained there is a centrally dictated number of hours for store tasks. They indicated the interventions would need to similarly be allotted hours for implementation and maintenance tasks. Similarly, central-management participants (P1-2a) emphasized that extra workload for stores should be limited. As a solution they proposed that product-specific signage could rotate between product groups over the year. This would limit workload at a specific time, make implementation and maintenance more efficient by focussing on a few shelfs, and follow participants’ belief that variation over time is necessary to keep signage impactful. Finally, the positioning of signage played a role in workload. Some spots were known to receive higher traffic, and thus require frequent maintenance of signage, adding to the workload, or risking a messy impression on customers. Such spots were abandoned for more sustainable alternatives.

###  Interactive

 This section focusses on interactive interventions, which are ‘the use of multi-media techniques seeking to draw attention, provide feedback on choices, or familiarise products, which depend on customer input or interaction.’ This was the most innovative group, and therefore discussions were more hypothetical. The major themes of discussion were maintenance, financing, customer habits, and technological limitations.

####  Maintenance

 For some interventions, maintenance was a barrier. Various participants (P2-3) expressed concerns regarding the more technical interventions. For the dynamic crate intervention, the machinery was perceived as difficult to maintain or repair, whereas for the gaze-nudge the risk of theft, or vandalism was a concern. *“Such a system isn’t bastard-proof” *[A2; central]. Due to both these concerns the dynamic crate intervention was dropped. To address the vandalism and theft barriers, it was agreed to explore ways to make the gaze-nudge more resilient, and better protected from theft (eg, a locked cover).

####  Financing

 Financing was another theme. The more ‘high-tech’ interventions incorporated expensive parts, whereas tastings required frequent oversight due to food-safety regulations, and possibly hiring a (perceived as more effective) specialised bureau. In P2-3 the funding of these interventions was discussed. It was agreed that one-time expenses for building the gaze-nudge would be covered by the project, and installation and electricity by the organisation. Tastings were complicated as their implementation by stores themselves would carry substantial workload, for relatively low expected effect, whereas external bureaus would need to be paid by the interventionists, who deemed this too expensive. Therefore, the idea of tastings was dropped in spite of initially positive feedback.

 “*I believe if you hire a professional bureau, then the effectiveness is very high. However, costs will also be very high” *[A2; central].

####  Customer Habits

 Store-management level participants in P2b believed the use of shopping lists to be highly habitual, and the people who would use them would have already made them before coming in the store. *“I believe it would be used very rarely” *[B5; store]. It was decided to focus on other interventions.

####  Technological Limitations

 Finally, there were plans to provide personalised feedback-messages on the receipts of customers. Unfortunately, the receipt system seemed unable to provide feedback messages in an adequately customisable format. Therefore, the design was dropped.

###  Constellation Elements and Dynamics

 The previous sections explored themes related to specific intervention types. However, several themes applied to a wide variety of intervention, indicating these represent more universal elements or dynamics in the supermarket-constellation.

####  Balancing Values

 The first theme is the balancing of commercial and health values. Participants would regularly indicate that, as a commercial organisation, commercial viability and success is a priority. *“You need to make a profit to be able to invest and keep existing as a cooperation” *[A6; central].This could be noticed in various discussions, where the effect of interventions on commercially important factors, eg, customer experience, efficiency, or profit margins, was an important consideration. This commercial value is also clear in structures such as the formula, planograms, and performance metrics, which guard efficiency and performance. This value, and related structures, often posed barriers, but occasionally facilitators, for sustainability.

 The organisation also emphasises social values. This is linked to the cooperative nature of the organisation, which is meant to serve the interests of its customer-members. The organisation frequently explores which societal/environmental issues are important to customers, and integrates these in their formula. This can be observed in the independent health-promotion initiatives organisational stores, as well as their engagement in our project. Currently, the stimulation of healthier diets is a major theme:

 “*We set out questions to a broad target group. This provided subjects which [ organisation ] finds important, and our stakeholders find important. (…) Stimulating healthy choices also came out of that as a focal point” *[A2; central].

 Participants indicated at various times that commercial and social values can seem in opposition, with commercial values seemingly holding priority. For example, promotions often feature unhealthy products because they were regarded as more profitable.

 “*I have mixed feelings, because healthy and unhealthy food, they are always perpendicular. And if you analyse where we sell most, it’s not the healthy stuff. So if I give priority to sales, that will include many unhealthy products”* [B5; store].

 However, participants also recognized the potential of health and commercial values to align, eg, improving public image to draw customers, or the high profitability of certain healthy products. To effectively serve both commercial and social values, opportunities need to be identified to align both:

 “*Yes, if it has added value, or the yield stays constant and there is added value in healthier diets and life - which is part of our formula, that we want to provide healthy and tasty food - then everyone will support this” *[A8; central].

####  Customer Experience

 As part of the commercial values, an important subtheme was the ‘customer experience,’ to retain customers. Participants perceived interventions which could be experienced negatively (judgemental, annoyance, or punishment), as undesirable or *“customer bullying” *[A1; central]. In response, the interventionists redesigned or dropped interventions to avoid customers having a negative experience, instead focussing on positive experiences (discounts, alternative suggestions, positive feedback).

####  Experiences and Beliefs 

 Throughout the design process, participants compared interventions to their experiences with similar practices, and from that basis formed believes on whether interventions would be effective at stimulating (healthy) product choices, and how easy or difficult it would be to implement interventions. Stronger familiarity, and positive previous experiences therefore seemed notable facilitators for open discussion, as the participant would be more willing to focus on the issue of sustainable design, rather than whether the intervention was worth developing at all.

 “*This is what we do all-out, we don’t do anything but create impulses. It’s simply marketing, that’s it. But in this case, it’s for a good cause” *[A14; central].

*“Yes, cards always work. When we have promotion cards on the shelves you see an effect from them” *[B5; store].

 Additionally, participants’ experiences could often prove valuable in designing the interventions to be more effective at stimulating healthy choices. Particularly on the topics of price, signage, product positioning, and dietary roles of products, participants had detailed ideas on how customers would react or could be engaged. Several of their suggestions were taken up in the intervention designs.

####  Trust in System

 Another theme was trust in organisational leadership, policies, and infrastructures. Participants at all levels seemed to default towards following the decisions higher-up in the organisation, with store-owners being especially reserved. Furthermore, we observed a strong tendency to follow organisational guidelines, metrics and systems, as translations of leadership decisions. This was also observed among store-owners, who trusted in the expertise of the management ingrained in these systems, and saw them as a tool for efficiency. This was regarded as a facilitator for the legitimacy of the intervention.

 “*I always assume that, if they thought about this carefully at the headquarters, then they thought about it better than I as entrepreneur would” *[B3; store].

## Discussion

 This paper addressed the research question “How does identifying and addressing sustainable-implementation barriers and facilitators through the use of a co-creation approach impact the process and outcomes of designing an HFI?” We found that a co-creation approach to the design of an HFI for a specific supermarket context helped us explore potential interactions between intervention designs and their implementation context. This allowed us to develop designs more likely to be sustainable and impactful in this context. This study contributes to the co-creation literature by exploring the benefits of its utilisation in the field of HFI design.

###  Quick Wins and Substantial Changes

 Based on our results, we hypothesise that the dynamics between HFI and implementation context can take various beneficial shapes. One is interventions which strongly align with the context. Another is interventions which push the boundaries of the context. Our reasoning is as follows:

 Interventions which were similar to existing practices, or otherwise aligned with constellation elements (signage, subsidies, symbols), were perceived as low-effort or cost, or even (commercially) beneficial, and therefore sustainable, by participants. These interventions are in alignment with their implementation context^5^: their relevant constellation elements are reconcilable. Therefore, such interventions can simultaneously meet intervention and implementation-context needs.

 Other interventions differed from existing practices (eg, dynamic crates), or went against constellation elements (eg, taxes, floor stickers), and were therefore regarded as unsustainable by participants. These interventions are in misaligned with their implementation context^5^: their relevant constellation elements differ in ways which make them irreconcilable, or in conflict. This means that the needs of intervention a context would come at cost for the other.

 Although it seems logical to equate ‘alignment’ with ‘feasible,’ and ‘misaligned’ with ‘unfeasible,’ our results suggest a more complex dynamic, where both have a place in co-creative design. We observed how aligned interventions served as ‘quick wins,’^35^ and built a sense of momentum and shared purpose between interventionists and supermarket actors. Cultivating such an environment is described, in systems innovation literature, as facilitative for change.^36,37^ In our design-process, aligned interventions (eg, positioning and signage) kept the process moving and built a foundation when other interventions (eg, taxes) encountered resistance, allowing us to revisit the latter at later times.

 Simultaneously, misaligned interventions present tools for impactful change: Unhealthy food-store environments are a product ‘unhealthy’ constellation elements. Therefore, changing those unhealthy elements would produce an inherently healthier food-store, compared to only addressing their ‘symptoms.’^38^ Misaligned HFIs are irreconcilable with constellation elements, which we hypothesise to be unhealthy, and implementing them would thus entail change to those elements. An example is our tax intervention, initially met with great resistance in spite of its potential effectiveness.^39^ Overcoming this resistance required continuous negotiation, and pushing of boundaries,^36^ illustrating the friction of misalignment. When an agreement was finally reached, this was a first step towards food-store organisations independently raising prices on unhealthy products, a major development towards accurately representing the societal health costs of these products in their prices.^40^

 Considering the previous observations together, we propose that aligned and misaligned interventions have complementary roles in co-creative HFI design, and could contribute towards addressing the systemic roots of unhealthy food-store environments. We believe the co-creative process was instrumental in utilising this dynamic, as the approach incorporates continuous, in-depth, and reflexive involvement of stakeholders, allowing for exploration of controversial topics.

###  Generalizability

 This study was conducted within the context of a Dutch supermarket chain, which may differ from other HFI contexts. However, the identified themes of barriers and facilitators seem consistent with those identified in our systematic literature review on HFIs in various retail contexts.^5^ Furthermore, other HFI studies similarly suggest that co-creative approaches may facilitate alignment between HFIs and their implementation context.^19,41^ This implies there could be value in the incorporation of co-creation approaches in future HFI-design, although more research in its application in various contexts is necessary.

###  Other Stakeholders for Scaling up

 This study within our project focussed on the design of interventions for a supermarket environment. Therefore, our participants were supermarket actors, as the future users of the interventions. Nevertheless, other stakeholders could have valuable input in certain stages of co-creative HFI design, particularly in relation to scaling up (both horizontal and vertical),^8^ such as roadmapping.^42,43^ Eg, another study in our project consulted consumers regarding their perspectives on HFI designs (Harbers et al,unpublisheddata), which revealed a variety of views: some appreciated the help in making healthier choices, whereas others expressed scepticism or distrust, particularly regarding the accuracy of health information and motives of the supermarket (Harbers et al, unpublished data). This distrust was in line with our findings and others literature,^44-46^ and indicates a need to involve consumers more strongly in the development of HFIs, which the potential added benefit of empowering them.^47^ In addition, in alignment with food-store participants’ views that their organisation lacked expertise on healthy food, a nutritional expertise centre was involved in the broader project. However, several participants proposed the involvement of trusted health authorities in the design and execution of the intervention. This would address the higher trust in dieticians, doctors, and governmental entities than academic and retailer parties.^45,48^

 Going beyond HFIs, and speculating about structural adaptations of the food-store system, governmental stakeholders could be included in the co-creative process to provide a level playing field while still promoting healthier products (eg, sugar-taxes). This has been suggested previously,^5^ and would facilitate commercially high-risk interventions such as price increases.

###  Strengths, Limitations, and Reflections

 The co-creative approach had several strengths. First, it engaged a wide range of actors, from a variety of backgrounds in the studied context. Second, the multiple feedback-cycles, along with involvement of new and recurring actors allowed for greater reflexivity and continuous development of the HFI interventions. Third, feedback from the interviews was checked with the interviewees to strengthen their validity. Finally, the fact that interviewees felt comfortable to express strong doubts and dislike implies there was little pressure for socially desirable answers, and the focus was on improving the HFI design.

 There were also limitations. First, we focused on a single organisational context, whereas multiple organisations could have provided additional perspectives. Nevertheless, we believe this paper illustrates the value of co-creation adequately. Second, our only participants were supermarket actors, whereas other stakeholders could be relevant. We believe that the current stage – initial design – is served by keeping the collaborating group compact, but concede that further stages, such as up-scaling, require the involvement of more stakeholders. As such, we are planning to engage industry, health, governmental, and public stakeholders, in our follow-up studies.

## Conclusion

 This paper presents the co-creative design process of an HFI in the context of a Dutch supermarket chain. The co-creation approach guided the collaborating stakeholders (interventionists and supermarket actors) towards an effective, sustainable, and up-scalable HFI design. Our results illustrate encountered barriers and facilitators, underlying mechanisms, solutions, and the involved considerations. The findings illustrate the potential of a co-creation approach for HFI design, and we reflect on the lessons learned regarding such methods and their improvement for future and broader use. We believe these insights to be valuable to interventionists and health policy-makers, as a tool to improve the sustainability, and therefore effectiveness and scalability, of HFIs.

## Acknowledgements

 This work was part of the SUPREME NUDGE project. Furthermore, we want to thank Femke de Boer, Jody Hoenink, Joline Beulens, Josine Stuber, and Lieke Vonk, for their assistance in the data collection.

## Ethical issues

 This study does not fall under the Dutch ‘Medical Research Involving Human Subjects Act,’ and therefore, official ethical approval was not required. We followed the ethical guidelines of the Faculty of Social and Behavioural Science of the VU University, Amsterdam.^49^ Before interviews, participants were informed of study design and goals, and their right to withdraw participation or sensitive information. Next, we asked consent to record and subsequently analyse the conversation which all participants agree to. Recordings, transcripts and the identification key were encrypted, and stored on a secure server, only accessible by the first and second authors. Transcripts were anonymized.

## Competing interests

 CNHM reports grants from Dutch Heart Foundation, grants from ZonMW, grants from NWO, during the conduct of the study; and the authors are currently in collaboration with the supermarket organisation described in this study. This being a co-creative study, the organisation was involved substantially in the study design and execution. Besides requesting the omittance of three instances of commercially sensitive information, the organisation made no attempts to structurally influence the content of this paper.

## Authors’ contributions

 CNHM: Conception and design, acquisition of data, analysis and interpretation of data, drafting of the manuscript. TJSW: Analysis and interpretation of data, drafting of the manuscript, critical revision of the manuscript for important intellectual content, supervision. JDM: Acquisition of data, critical revision of the manuscript for important intellectual content, obtaining funding, supervision. JEWB: Drafting of the manuscript, critical revision of the manuscript for important intellectual content, obtaining funding, supervision.

## Supplementary files


Supplementary file 1. Interview Guides, and Descriptions of Discussion Pieces and Data Collection Tools. This file contains, in chronological order, interview guides, and descriptions of the discussion pieces and data collection tools, used as part of our data collection. Interview guides have been translated from their original language (Dutch) to English.

Supplementary file 2. Overview of Barriers and Facilitators Per Intervention. This table provides an overview of the barriers and facilitators for each specific intervention, and the associated barriers and facilitators which manifest from these themes.

Supplementary file 3. Overview of Barriers and Facilitators Related to Supermarket-Organisation Constellation Elements. This table provides an overview of the constellation elements, their relevant subthemes, and the barriers and facilitators that arose from each of them.
