# Supplementary material for: Designing a Healthy Food-Store Intervention; A Co-Creative Process Between Interventionists and Supermarket Actors
Source: Int J Health Policy Manag. 2021 Sep 11;11(10):2175–88. doi: 10.34172/ijhpm.2021.110 (PMC9808264; doi:10.34172/ijhpm.2021.110)
Supplement: Supplementary file 1 — Interview Guides, and Descriptions of Discussion Pieces and Data Collection Tools. This file contains, in chronological order, interview guides, and descriptions of the discussion pieces and data collection tools, used as part of our data collection. Interview guides have been translated from their original language (Dutch) to English. [file ijhpm-11-2175-s001.pdf]

Article title: Designing a Healthy Food-Store Intervention; A Co-Creative Process Between Interventionists and Supermarket Actors

Journal name: International Journal of Health Policy and Management (IJHPM)

Authors' information: Cédric N.H. Middel[1]*, Tjerk Jan Schuitmaker-Warnaar[1], Joreintje D. Mackenbach[2,3], Jacqueline E.W. Broerse[1]

[1]Athena Institute, Faculty of Science, Vrije Universiteit Amsterdam, Amsterdam, The Netherlands.

[2]Department of Epidemiology and Data Science, Amsterdam University Medical Centers, Vrije Universiteit Amsterdam, Amsterdam, The Netherlands.

[3]Upstream Team, Amsterdam University Medical Centers, Amsterdam, The Netherlands.

(*Corresponding author: c.n.h.middel@vu.nl)

Supplementary file 1. Interview Guides, and Descriptions of Discussion Pieces and Data Collection Tools

IGp1 (interview guide phase 1)

Translation from original language (Dutch)

Personal introduction, explain the study, ask for informed consent

Part 1: Actor and organisation

1. What is your current work in the organisation?
    a. **Probe**: How would you be involved in our interventions, if they were put into practice?
2. Can you tell me how your department functions?
    a. **Probe**: Why is the department set up in this way?
3. Which people/departments/external organisations do you interact with?
    a. **Probe**: What are these interactions like?

Part 2: Systemic problem

1. What is your view on health promotion in the Dutch supermarket sector?
    a. **Probe:** What are the causes of this problem?
    b. **Probe:** How could this problem be resolved?
    c. **Probe:** Why do you think your organisation is involved in this collaboration?

Part 3: Intervention

1. **introduce verbal examples of four intervention ideas* [BROAD IDEAS]
2. *Per ideas:* What do you think of this approach? *(focus primarily on those close relevant participant's function)*
    a. **Probe**: Do you perceive any barriers/facilitators?

        i. **Probe**: How can we work around this barrier?
        ii. **Probe**: How can we utilise this facilitator?
    b. **Probe**: Do you have ideas to change/improve this approach?
    c. **Probe**: Do you have any suggestions on how to develop this approach?

Conclude the interview

Broad Ideas (discussion piece phase 1)

The discussion piece for phase 1 consisted of a list of broad ideas for interventions (see table 1). The ideas were divided into four groups: 1) Price strategies, 2) Signing & communication, 3) Positioning & presentation, and 4) Interactive & experimental. Each group was briefly described and explained, and examples were provided.

IGp2a (interview guide discussions phase 2a)

Translation from original language (Dutch)

Personal introductions, explain the study if participant is new, ask for informed consent

Parts 1 and 2 are repeated for each of the listed nudges/price strategies

1. *Provide* [PROTOTYPE GUIDE] *to the participants, reiterate which intervention type will be discussed in this group (either 'prices' or 'nudges').*

2. *Discuss the relevant sections (targeted products, intervention designs, conditions). The discussion is semi-open: these topics need to be covered:*
    a. **Topic**: Do you consider the intervention feasible to implement?
        a. (*if not)* why?
        b. Could we adjust the design to resolve this problem? Or develop a workaround?
            i. *Seek a mutually acceptable solution*
                1. *If found, note this down for part 3*
                2. *If not found, discuss how to proceed with the development of this intervention*
    b. **Topic**: Do you consider the intervention acceptable to implement?
        a. (*if not)* why?
        b. Could we adjust the design to resolve this problem? Or develop a workaround?
            i. *Seek a mutually acceptable solution*
                1. *If found, note this down for part 3*
                2. *If not found, discuss how to proceed with the development of this intervention*
    c. **Topic**: Do you believe the intervention would be effective at health promotion?
        a. (*if not)* why?

b. Could we adjust the design to resolve this problem? Or develop a workaround?
    i. *Seek a mutually acceptable solution*
        1. *If found, note this down for part 3*
        2. *If not found, discuss how to proceed with the development of this intervention*

3. *Make a summary of the discussed solutions*:
    a. Are any important adjustments missing?
        i. If not: discuss
    b. Are any important issues with the interventions not discussed?
        i. If not: discuss
    c. Do we agree on these adjustments that need to be made to the intervention designs?
        i. If not: discuss

Conclude the interview

Prototype Guide (discussion piece phase 2a)

Table 1 describes the proposed nudges and pricing strategies to be implemented as fixed or varying intervention components, targeting different products in product groups. Choices for specific targeted products were based on the Dutch nutrition guidelines, and a detailed explanation was provided to the supermarket-organisation. In addition, we provided scientific evidence for the proposed intervention components based on previous literature. We summarised this information in tabular form and an accompanying report.

Table 1. Overview of proposed interventions per product group

	**Fruits and Vegetables**	**Grain products**	**Legumes**	**Fats**	**Beverages**	**Nuts**	**Fish**	**Dairy**	**Meat (and replacements)**	**Sweets, snacks, chips, cookies**
					**SWITCHING NUDGES**					
**Healthy check-out**	X									
**End-of-aisle shelves**		X	X	X	X	X				
**Basket/ second placement**	X	X	X		X					
**Meal suggestions**	X	X	X							
**Product bundling**	X	X	X							
**Frames**	X							X		
**Shelf talker**	X	X	X	X	X					
**Shelf tags**	X	X	X	X	X	X	X	X	X	
**Arrow signage**	X	X	X	X	X	X	X	X	X	
**Colour/shape-organisation**	X									
**Tasting**	X									
					**CONSTANT NUDGES**					
**Floor signage**	X	X	X							

**Cart handles**										
**Cart signage**	X	X	X							
**Images of products**	X	X	X							
**Posters**	X	X	X							
**Shelf positioning**		X	X	X	X	X	X	X	X	X
**Visualising product content**					X					
**Default nudge**		X								
**Dynamic nudge**	X									
**Feedback**			X							
**Functionality**										
**Dimming lights**										X
**PRICE STRATEGIES**										
**Tax**		X		X	X	X		X		
**Subsidy**	X	X	X	X	X	X	X	X		

IGp2b (interview guide phase 2b)

Translation from original language (Dutch)

Personal introduction, explain the study, ask for informed consent

Part 1: Interviewee

- Ask participant to introduce themselves
- Can you tell us about:
  - your experience as supermarket manager/owner
  - characteristics of the client base
  - relationship with Coop
  - (personal) affinity with health

Part 2: Intervention

- *Discuss, in sequence, the interventions* [SUMMARY]
  - Would the intervention be feasible in your store?
    - Why (not)?
    - What could be improved, and why would this be an improvement?
  - Would the intervention be effective at promoting healthier choices in your store?
    - Why (not)?
    - What could be improved, and why would this be an improvement?
  - Would you want to implement the intervention in your store?
    - Why (not)?
    - What could be improved, and why would this be an improvement?

Part 3: Own input

- Do you have any ideas of your own that you feel would be effective at stimulating healthier choices among your customers?
- Why do you feel this would be effective, feasible, and desirable?

Conclude the interview

Summary (discussion piece phase 2b)

The discussion piece for phase 2b consisted of a condensed summary of proposed interventions, describing shortly what each should physically look like in the environment of a supermarket (see table 2 for an overview).

Table 2. Overview of interventions discussed with the interviewee

Intervention type	Intervention
Positioning & presentation	Baskets/second placement products
	Shelf positioning products
	Meal suggestions
	Ordering fruit and vegetables shelves on colour
Signage & communication	Floor signage
	Communication on shopping carts/baskets
	Shelf signage (arrows, frames, cards)
	Visualising calory/sugar content products
Interactive & experimental	Dynamic nudge
	Stimulating shopping lists
	Tasting
Price strategies	Tax
	Subsidy

IGp3 (interview guide phase 3)

Translation of original (Dutch)

Introduction (10 minutes)

- Intro talk, explanation about SN, workshop set-up, and group distribution
- Participants take place in one of three groups. Group leaders check whether the functions represented fit the topics the group will discuss.

Group Assignment: (60 minutes)

- Groups receive one of three [EXTENDED SUMMARIES]
- Groups draw a [SCORING MATRIX]
- The group takes 1-5 minutes, depending on the number of interventions to be discussed, to read the explanation of an intervention, and to discuss any uncertainties. Subsequently, the intervention is written on a yellow post-it, and there is a discussion about where in the matrix the component should be placed. This is repeated until all interventions assigned to the group are discussed and placed.
    - **Probe:** Why does an intervention score high / low on an axis?
    - **Probe:** Is this inherent to the intervention, or an external factor?
    - *Difficulty getting consensus within the group? Then leave it open for the plenary discussion.*
- If the component scores + / +, this implies it could be implemented as-is. Verify this with the group.
- If the component falls into one or both - areas, it has some issues. Ask the group what conditions must be met to get the component to the + / + area.
    - Write these conditions on a new post-it (distinct colour), with the name of the component, and place it at the position the component would belong if this improvement was realised.
        - **Probe:** What needs to be changed?
        - **Probe:** Which stakeholders are needed for this?
        - **Probe:** What will this cost, and is that cost worth the outcome?
        - **Probe:** How realistic is this?
- **Each group discusses what they consider the most important interventions (and their conditions) to invest in. This will be presented to the other groups.**

BREAK-- 10 minutes --

Plenary Discussion (50 minutes)

- Each group briefly explains how they scored the interventions, and what they think the key points from their discussion.
    - Which scored high on both areas (+/+)?
    - Which scored low on one or two areas, but could be improved to score high?
        - Which conditions would need to be met in this case?
- When a group has presented, the other groups can respond. The facilitator keeps track of the outcome of the discussion on a new matrix.

Conclude the workshop

Extended Summaries (discussion piece phase 3)

The discussion pieces for phase 3 consisted of an extended summary of all proposed intervention components. This was split up into three versions, one of which focussed primarily on product positioning and presentation, another primarily on signage and other forms of communication, and the final one on price strategies. Interactive & experimental interventions were divided across the first two groups (see table 3 for an overview). Each summary consisted of the intervention names, short descriptions of what interventions should physically look like in the environment of a supermarket, previously agreed upon boundaries and guidelines for their designs and implementation processes, and finally some of the alternatives to its design or implementation process (as suggested by participants in previous phases).

Table 3. Overview of interventions discussed by each workshop group

Workshop group	Interventions discussed
1 Signage & communication	Symbols
	Small shelf signage
	Large shelf signage
	Frames on cooling doors
	Shopping carts/baskets signage
	Product images
	Gaze nudge
	Feedback signage
	Savings promotion
2 Positioning & presentation	Symbols
	Healthy check-outs
	End-of-aisle shelves
	Baskets/second placement products
	Shelf positioning products
	Meal suggestions
	Tasting
	Floor signage
3 Price strategies	Tax
	Subsidy

Scoring Matrix (tool phase 3)

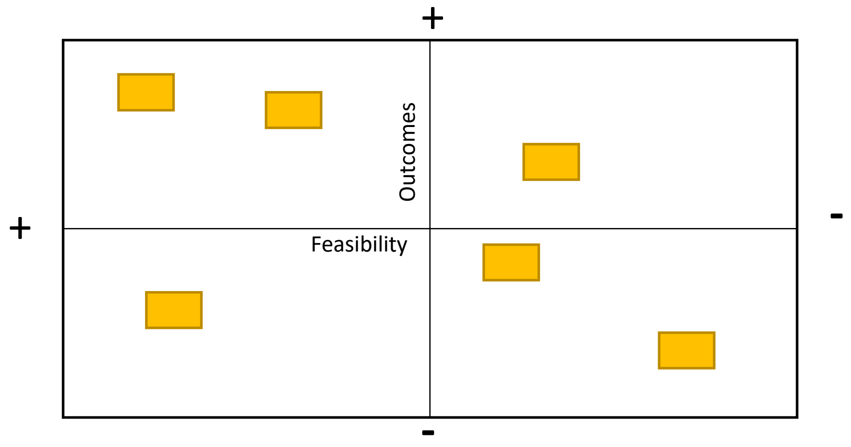

- The Y-axis represents the combined value of the expected outcomes (financial, commercial, social, health)
- The X axis represents the combined feasibility of the intervention in its implementation context (weighing barriers and facilitators, complexity, resources needed).
- Participants write down each intervention on a post-it, and place the post-it on the matrix, in accordance to their expectations regarding the outcomes and feasibility.
- If the component scores negative on one or both axes, participants can write possible improvements for the component on a new, different coloured, post-it, along with the name of the intervention. This 'Improved' post-it is placed in the appropriate position for the scenario where this improvement is realised.

Important is that participants are asked to explain each decision in this exercise. The facilitator should always ask why an intervention receives a certain score, and why and how proposed improvements lead to a different score.
