# Supplementary material for: Designing a Healthy Food-Store Intervention; A Co-Creative Process Between Interventionists and Supermarket Actors
Source: Int J Health Policy Manag. 2021 Sep 11;11(10):2175–88. doi: 10.34172/ijhpm.2021.110 (PMC9808264; doi:10.34172/ijhpm.2021.110)
Supplement: Supplementary file 2 — Overview of Barriers and Facilitators Per Intervention. This table provides an overview of the barriers and facilitators for each specific intervention, and the associated barriers and facilitators which manifest from these themes. [file ijhpm-11-2175-s002.pdf]

Article title: Designing a Healthy Food-Store Intervention; A Co-Creative Process Between Interventionists and Supermarket Actors

Journal name: International Journal of Health Policy and Management (IJHPM)

Authors' information: Cédric N.H. Middel[1]*, Tjerk Jan Schuitmaker-Warnaar[1], Joreintje D. Mackenbach[2,3], Jacqueline E.W. Broerse[1]

[1]Athena Institute, Faculty of Science, Vrije Universiteit Amsterdam, Amsterdam, The Netherlands.

[2]Department of Epidemiology and Data Science, Amsterdam University Medical Centers, Vrije Universiteit Amsterdam, Amsterdam, The Netherlands.

[3]Upstream Team, Amsterdam University Medical Centers, Amsterdam, The Netherlands.

(*Corresponding author: c.n.h.middel@vu.nl)

Supplementary file 2. Overview of Barriers and Facilitators Per Intervention

Supplementary File 2: Overview of Barriers and Facilitators Per Intervention. *This table lists the interventions designed through the co-creative process, classified in broader classes. The right column lists facilitators (+), barriers (-), and neutral or mixed (●) factors relevant to the implementation and up-scaling of these interventions. The middle column indicates for which interventions these factors appeared to be relevant.*

Intervention Class	Intervention			Associated Facilitators (+) & Barriers (-)
**Price strategies** Price increases and decreases to deter unhealthy and stimulate healthy choices.	**Subsidy**	**Tax**		
	X	X	+	Low-income consumers are perceived as highly sensitive to prices, leading to high expectedeffectiveness.
	X	X	+	Most targeted product groups contain both unhealthy products and feasible healthier alternatives, to allow feasible inter-group 'switching'. This also opens up the option to have taxes and subsidies in one group compensate each other to remain cost-neutral.
	X	X	+	High curiosity on the outcomes of such an experiment.
	X		+	Strong similarity to existing discount practices gives impression of feasibility.
	X	X	+	High-level decision-makers supported the idea.
		X	+	Due to the limited initial scope of the intervention, financial and image risks were seen as minor, and high-level support led to generall acceptance of the idea.
		X	+	Agreement with a major authority on supermarket-chain price-comaprisons, to exclude intervnetion pilot stores from comparisons.
	X		+	Perceibed as likely to be appreciated by customers, for either the health stimulation or financial benefit, and therefore improvement to chain image.
	X		+	Fits with the existing practice of discounts as a marketing tool.
	X	X	–	Minor scepticism regarding the (long-term) effectiveness of component in changing diet habits.
	X	X	–	Potential risk of financial losses due to reduced profit-margins (subsidy), or reduced sales-volume (tax). Furthermore, unhelathy products are often regarded as more profitable.
	X	X	–	Need to carefully consider between which products customers will likely switch to healthy alternative when price differences are itnroduced, and for which they will simply go to the competitor.
		X	–	Organisational taboo on the topic of price increases leads to general resistance.
		X	–	Potential to frustrate customers, possibly leading to conflicts, or driving them to other chains. Requires a clear and strong explanation.
		X	–	Increasing prices perceived as unfairly punishing customers, not allowing 'free, unjudged, choice'.
		X	–	Fear of seeming more expensive compared ot other chains in media price-comparisons.
	X		–	Limited profit-margins on products limit subsidy options.
	X	X	•	Store prices are centrally managed, and this system not suited for longer-term local differences, as needed for these components. This leads to manual implementaiton and high additional workload. The rollout of the a system is coming, which is suited for local price-differences. However, this process has some delays.
	X	X	•	Potential risk of financial losses due to reduced profit-margins (subsidized products), or reduced sales-volume (taxed products). Alternatively, potential financial benefits due to increased sales volume (subsidy) and greater profit margins (tax).

**Presentation & positioning**	**Shelf positioning**	**Second placement**	**Meal suggestions**	**Healthy check-out**	**Scarcity nudge**	**Default nudge**	
**Adjustments in product positions, availability, or presentation on shelfs, or presentation of healthy products in alternative places through the store.**	X	X	X	X		X	+ **Positive expectations of the impact of components on dietary behaviour, often due to perceived strong ability to draw attention towards the promoted products.**
	X	X	X	X		X	+ **Perceived as generally easy or low-effort/cost to implement due to similarity/integratability with current marketing practices and systems.**
	X	X	X				+ **High similarity to existing positioning and marketing practices leads to openness to the ideas and well-supported feedback on feasibility and effectiveness.**
	X	X					+ **Shelf position matrixes (planograms) are centrally coordinated and maintained, and integrated in the resupply system. This makes local deviations highly labour intensive, which strengthens compliance with the planograms in place. Components could be integrated as separate planograms.**
	X	X					+ **The usual products used for second placement include healthy options, which can be focussed on in intervention stores. Furthermore, most product groups contain both unhealthy products and feasible healthier alternatives to promote inter-group switching.**
		X					+ **There are some options for unusual secondary presentation spaces which can accomodate products not usually presented in such capacity.**
		X					+ **Perceived to increase sales, and therefore profits, of promoted products.**
		X	X	X			+ **Several options exist and are feasible to adapt the presentation spaces to accommodate the components and unusual products.**
				X			+ **Adjusted presentation space presents usually sees high sales among target group.**
				X			+ **Expectation of positive/appreciative reactions by customers to this component.**
				X			+ **Willingness among high-level decision-makers to take some fianncial risks for experiemntation with new ideas**
					X		+ **Perceived as interesting and novel. Curiousity to its outcomes.**
	X	X	X	X	X	X	– **Negative expectations of the impact of components on dietary behaviour, often due to perceived weak ability of component to draw attention towards the promoted products, expectations that effects will take time to occur, and concerns wether some healthy alternatives are too far seperated in the store to present a feasible [illegible]**
	X	X		X		X	– **General fear of financial losses, and strong desire to closely monitor outcomes and possible adjust/cancel components.**
	X	X					– **May go against organisational policy of no/as-little-as-possible local deviations in presentation and positioning, and focus on an enjoyable, succesfull, and effortless shopping experiences for customers**
			X	X			– **Store may not have these specific presentation spaces, or the space may not be able to carry the desired healthy products.**
	X	X	X	X	X	X	– **May go against organisational focus on an enjoyable, easy, and peacefull shopping experiences for customers. Somtimes perceived as annoying (having to search for products) or overwhelming (high number of additional presentations in the store) for customers.**
	X	X				X	– **Health stimulating goal may not be clear to customers without explanation**
	X	X					– **Planograms are labour intensive to develop and maintain, and capacity for additional ones for components is limited.**
	X	X				X	– **Component may impose additional workload onto store staff, which is already high**

	X						– Stores can use different planograms for the same productgorup due to size differences, meanign the adjustment of one group on the shelves can require multiple new planograms.
		X	X	X			– Floor-and secondary presentation spaces are limited and highly contested in stores. These are ususally reserved for specific products (e.g. weekly promoted items). Presentation spaces may also not be able to carry the desired healthy products.
		X		X			– Products have a variety of presentation requriements (e.g. temperature, shape, humidity), which are not always compatible.
					X	X	– Perceived as an obstruction/annoyance for customers, and therefore detrimental to their shopping experience, possibly driving them to go to a competing chain.
	X	X	X	X			– Lack of organisaitonal knowledge on what qualifies as a healthy product and thus should be promoted.
	X					X	– Products put into less desirable position may negatively affect their contribution to store finances, and negatively affect relations with their producers.
			X				– Customers may be sceptical towards health-related claims made by supermarkets.
		X		X			• Mixed perspectives on wether healthy products can be similarly profitable to unhealthy ones in the spots used by these components.
**Signage**<br>**The use of visual media throughout the store or near products to draw attention, communicate a theme, or provide general or specific information or feedback.**	**Symbols**	**Shelf signage**	**Door signage**	**Large signage**	**Cart/basket signage**	**Floor/ ceiling signage**	
	X	X	X	X	X		+ High similarity to existing positioning and marketing practices leads to openness to the ideas and well-supported feedback on feasibility and effectiveness.
	X	X	X	X	X		+ Positive expactations of the impact of components on dietary behaviour, often due to perceived strong ability to draw attention towards the promoted products, and previous positivce experiences with similar practices.
	X	X	X	X	X	x	+ Perceived as generally easy or low-effort/cost to implement due to similarity/integratability with current marketing practices.
	X	X	X	X	X	X	+ Components are expected to be effective at driving sales of promoted products, leading to financial benefits.
	X	X	X	X			+ There is broad organsiational support for the new signage policy, and designing components to fit with it is highly feasible. This could drive easy acceptance of components and utilise the existing knowledge ingrained in the policy.
	X		X	X	X	X	+ Due to similarity ot existing practices, components are perceived as easy, efficient, or low-cost, to carry out
	X						+ Non-health related signage was found to fit closely in existing product values focussed on by organisaiton.
	X	X	X	X			+ Expectation of positive/appreciative reactions by customers to this component.
	X	X		X			+ Components are perceived to fit the interests or shopping behaviours of the target group
		X					+ Targeted product groups include both unhealthy products and healthy alternatives, for signage to feasibly stimulate a shift between..
	X					X	+ Perceived as interesting and novel. Curiousity to its outcomes.
		X	X	X	X		Feasible options regocnized by interviewees to adapt shelves and presentation spaces to accommodate unconventional signage.

	X	X	X	X	X	X	– Negative expectations of the impact of components on dietary behaviour, due to similarity with existing or historical practices, or personal experiences, which showed such methods as ineffective or unfeasible. Only floor/ceiling signage was viewed as negative by a substantial majority.
	X	X		X	X		– Components perceived to possibly lead to financial losses due to negative shopping experiences, or limiting sales of high-profit products.
	X	X	X	X	X	X	– According to interviewees experiences, signage has an optimum frequency. When it becomes too much, customers will mentally block it out and not react anymore.
	X	X	X	X	X	X	– The organisation has a strict policy on in-store signage. This is a minimalistic, less-is-more approach which is widely supported. Components should be designed in accordance with this policy.
	X	X	X				– Due to their high level of autonomy, there were concerns regarding the fidelity of privately-owned stores in maintaining component.
	X	X	X	X	X	X	– Too much signage is believed to be overwhelming to customers, and make the store look less appealing.
	X	X	X		X		– Signage is believed to have diminishing returns in it s ability to draw attention. Signage not aimed at providing information should be switched around frequently.
		X		X	X	X	– Some forms of signage, or certain positions are likely to fall down, get dirty, or break quickly, and lead ot frequent messes.
		X	X	X			– Signage can block the view or product access for customers, which is undesirable.
	X	X	X	X	X	X	– Lack of organisaitonal knowledge on what qualifies as a healthy product and thus should be promoted. Furthermore, there are cocnerns regardign the wrongfull promotion of unhealthy products, as potentially damaging to public image.
	X	X	X	X	X	X	– Customers may be sceptical towards health-related claims made by supermarkets.
		X		X			– Texts on signage regardign unhealthy products may come across as patronizing or judgemental, which negatively affects the shopping experience
	X	X	X		X		– Putting up product-specific signage and frequently correcting it when products move was perceived as labour intesive for the stores, which already have limited work-hours and staff.
		X					– At times, industry representatives may put up their own signage in stores, which may block out or intervere with components.
	X	X					– Signage indicating that some products are healthy may imply to custoemrs that others are not, and turn them away from buying those. This was regarded as potentially neglecting important parts of the product range, and possibly harmfull ot industry relations.
		X		X	X		– Information presented by this component was perceived as not of interest to the target group.
	X						– Component likely requires explanation for customers, which will take time and limit its initial effectiveness.
					X		– Carts/baskets vary between stores and are not always suited to carry signage.
				X			– Stores have limited space available to place larger forms of signage.
				X			– Folders/flyers are usually heavily subsidized by the industry, in return for prominent placement of their products. Component would not be able to be financed in the same way, making it costly.

	X	X	X	X	X	X	• Divisive opinions on wether certain components would be noticed/read by customers or not. Generally, most opionions were positive regarding the more developed and adjusted versions of each component, with the exception of floor/ceiling signage.

**Interactive** The use of multi-media techniques seeking to draw attention, provide feedback on choices, or familiarise products, which depend on customer input or interaction.	**Tastings**	**'Gaze-nudge'**	**Feedback receit**	**Dynamic crates**	**Shopping list materials**	
		X		X		+ Component seen as innovative and an interesting experiment to try out.
	X	X			X	+ Component expected to incite positive reactions from customers, and being appreciated or seen as fun or exciting.
		X		X		+ Component perceived to be effective at drawing attention customers.
	X					+ Component believed to stimulate greater sales, for financial benefits.
		X		X		– Component expected to incite negative reactions from customers, possibly scaring them, or block access to products.
	X	X		X		– Component regarded as high-effort implement and maintain, often due to health regulations, staff needed, being annoying for staff in their regular practices, or the potential for vandalism or theft.
	X	X		X		– Component expected to be expensive to build or execute properly due to materials needed, or experienced staff requried.
	X				X	– Component perceived as unlikely to be used by customers, making it redundand.
			X			– Component not possible under current check-out system.
	X					– Component limited in products it can promote by the differing preperation and preservation requirements for products (mainly temperature and air exposure).
				X		– Crates would likely get dirty fast due to humidity of fruits and vegetables section.
	X	X			X	– Component perceived to be unappealing to targetgroup.
		X				– Component perceived as possibly judgemental or condescending to customers.
		X				– Component is expected to have diminishing returns in terms of ability to draw customer attention and stimulate healthier dietary choices.
	X	X	X	X	X	• Mixed opinions regarding the effectiveness of components for stimulating healthy dietary choices. Generally itnerviewees found it difficult to make a strong prediction. Tastings were generally perceived as effective IF carried out in a well-financed manner.
