# Supplementary material for: Designing a Healthy Food-Store Intervention; A Co-Creative Process Between Interventionists and Supermarket Actors
Source: Int J Health Policy Manag. 2021 Sep 11;11(10):2175–88. doi: 10.34172/ijhpm.2021.110 (PMC9808264; doi:10.34172/ijhpm.2021.110)
Supplement: Supplementary file 3 — Overview of Barriers and Facilitators Related to Supermarket-Organisation Constellation Elements. This table provides an overview of the constellation elements, their relevant subthemes, and the barriers and facilitators that arose from each of them. [file ijhpm-11-2175-s003.pdf]

Article title: Designing a Healthy Food-Store Intervention; A Co-Creative Process Between Interventionists and Supermarket Actors

Journal name: International Journal of Health Policy and Management (IJHPM)

Authors' information: Cédric N.H. Middel[1]*, Tjerk Jan Schuitmaker-Warnaar[1], Joreintje D. Mackenbach[2,3], Jacqueline E.W. Broerse[1]

[1]Athena Institute, Faculty of Science, Vrije Universiteit Amsterdam, Amsterdam, The Netherlands.

[2]Department of Epidemiology and Data Science, Amsterdam University Medical Centers, Vrije Universiteit Amsterdam, Amsterdam, The Netherlands.

[3]Upstream Team, Amsterdam University Medical Centers, Amsterdam, The Netherlands.

(*Corresponding author: c.n.h.middel@vu.nl)

Supplementary file 3. Overview of Barriers and Facilitators Related to Supermarket-Organisation Constellation Elements

This table provides an overview of the themes identified within our application of the theoretical framework, and the associated barriers and facilitators which manifest from these themes. Barriers and facilitators are preceded with a '-' and '+' respectively, with a '●' indicating phenomena which can be interpreted as both. The classification following each barrier/facilitator indicates in which phase(s) of the design process they were encountered, the numbers correspond to the phase, whereas [A] indicates presence across all phases.

Domain	(Sub)Themes		Associated Barriers & Facilitators
Structuring Elements [Culture]	Commercial values and priorities	Commercial viability<br>• High value was placed on the commercial viability and health of the organisation.<br>• Store owners are directly dependent on turnover for their income.<br>• For these reasons, intervention components were approached as a business case, where balance between costs/risks and benefits is important.	- Perceived commercial risks/costs, including: reduced sales (unhealthy products), reduced profits, increased waste, losing customers. [A]<br>+ Perceived commercial benefits, including: higher sales (healthy products) increased profits, drawing customers. [1/2b/3]
		Innovation/experimentation<br>• Experimentation and innovation were considered important for continued competitiveness, and worth small risks for long-term improvement.	• Price increases were initially taboo due to high associated commercial risks [1/2]. In later stages actors became more open to the idea [2a].<br>- Actors desired organisational monitoring and control over experiments, to contain negative commercial outcomes early on. [2/3]<br>+ General openness to and valuing of explore novel ideas. [A]<br>+ General willingness to take small commercial risks in order to learn. [2/3]

		**Customer experience & perception**<br>• High value was placed on how customers experience their shopping trip, and their perceptions of products. Positive experiences and perceptions were believed to stimulate customer spending and loyalty to the chain.	- Component may lead to negative store experience [1/2], by making the store seem messy, unhygienic, or chaotic [1/2b/3], making customers feel judged/punished [1/2b/3], or causing inconveniences in the shopping trip [A].<br>- Others were perceived to negatively affect how customers perceive certain products. [A]<br>+ Some intervention components, e.g. information or interactive components, were expected to improve customer experience. [2b]
		**Public perception**<br>• Public perception of the organisation was considered important to draw in new customers and retain existing ones.<br>• The truthfulness of health claims was considered very important, to ensure a trustworthy image and avoid potential backlash later on.	- Concerns about making health claims which could be proven wrong later, harming public image [1/2b/3].<br>- The public was perceived to distrust health claims made by the organisation or perceive an ulterior motive. [1/2a/3]<br>- Price increases were considered harmful for public perception, by making the chain seem expensive compared to competition. [1/2]<br>+ The positive health goals of intervention components are perceived to possibly outweigh the negative aspects of components for the public. [3]
	Social values and priorities	**Meeting customer interests**<br>• It is considered an organisational priority to serve the needs and interests of customers. This may positively impact public image.	+ Health is of (growing) interest to customers. [A]<br>- But these interests can change over time. [1/2b]
		**Health**<br>• Health is currently an organisational interest, likely because of it being a customer interest.<br>• Various commercial benefits are perceived to health promotion, including matching customer interests, increased longevity of customers, and improved public image.	+ Health is considered an issue of importance in the organisation, particularly among central management where it's institutionalised in 'corporate social responsibility policies', but also for separate store owners/managers. {1/2b]
	Management values and priorities	**Hierarchy**<br>• The organisation has a robust hierarchy. Opinions and approval of management are considered important. Organisational policies are closely followed.<br>• An exception are independently owned stores, where owners seem relatively free.<br>• Trust in the expertise of central management and systems is high, meaning even independent store-owners often follow standard practices.	- The independence of privately owned stores can lead to lower adherence to intervention plans. [1/2a/3]<br>+ Store managers will follow the directions of central management, store owners usually follow major policies as well. [A]

	Beliefs	Experiences & intuition<br>• Many prevalent beliefs were based on previous experiences from actors. Evidence varied in quality, between anecdotal, usually concerning behaviour, to seemingly robust analysis and predictive models for product sales and supply chain.<br>• Beliefs were commonly tied to comparable current or past practices in the supermarket sector.<br>• In cases where there were no experiences to draw upon, intuition was followed. Actors often reflected that this had no real evidence basis.	- Some components were regarded as similar to current/past practices which had negative experiences. [A]<br>+ Some components were regarded as similar to current/past practices which had positive experiences. [A]<br>• Some components were liked or disliked based on 'gut feeling'. [A]
		Beliefs on shopping behaviour<br>• Some variety in these beliefs was observed, but general ideas were consistent.<br>• Choices were believed to be habitual or impulsive, and based on perceptions of price, convenience, quality, and taste.<br>○ (Perception of) price was often believed to be the greatest influence in choice, especially among lower-income groups.<br>• People were generally believed to prefer unhealthy products, due to taste and perception as cheaper. However, a trend of growing interest in healthy products was perceived in some areas.<br>• Beliefs regarding the influenceability of shopping behaviour varied. Generally, shopping was perceived as strongly habitual, and thus difficult to change, but it could be steered through marketing methods. Especially impulsive choices were regarded as steerable.<br>○ Some also believed customers were only open to influence up to a point, after which their choice was set.<br>○ Theories also existed regarding where customers look during their shopping trip. Anything outside these spot in space and time is unlikely to influence them.	- Generally, unhealthy products were perceived as more popular than healthier ones. [A]<br>+ A societal trend of growing interest in healthy products is observed. [A]<br>+ Shopping behaviour is perceived to be steerable through various methods, some of which are similar to the proposed intervention components (presentation, promotion, pricing, information). [A]
		Intervention cost/benefit beliefs<br>• There were multiple beliefs regarding the potential costs and benefits of components with regards to required resources, and resulting impact on health and consumer behaviour, or commercial metrics. These could vary substantially between actors.<br>• These beliefs were often based on general perceptions regarding which marketing methods should be applied where and how. Specific methods were believed to fit specific roles, and specific spots were believed to be effective or not.<br>• Components were generally judged based on perceptions of similar marketing methods or phenomena. If these did not exist, intuition was leading.	- Some components were believed to have low potential impact, or innovative value, often supported by negative experiences or intuitions. [A]<br>○ A subcategory here is the belief that certain components are less likely to be seen by customers, or applied on easily overlooked places. [2/3]<br>- Customers are believed to only have a limited capacity to interpret signage and other signalling in a store, leading to a filtering of any non-essential information, and thus diminishing returns in impact. [A]<br>- Some components were believed to have high implementation costs in terms of workload or money. [A]

			+ Some components were believed to have high potential impact, or innovative value, often supported by positive experiences or intuitions. [A]<br>o These components were generally believed to be more visible to customers, or applied on easily spotted places. [2/3]<br>o High coherence between components was believed to facilitate greater impact on customers. [2b/3]<br>+ Some components were believed to have lower costs in terms of workload or money. [A]
		Product beliefs<br>• All products in the store are believed to fulfil a certain role. Some are meant to provide profits (high profit-margin/volume sellers), others are there to draw in customers (promoted A-brands), or meet a customer need (toilet paper).<br>• The demand was generally believed to be highest for cheaper unhealthy products.	- Some components did not fit with the role of targeted products, making them ineffective. [A]<br>- Due to their higher demand, unhealthy products were generally perceived as more profitable. [A]<br>+ Due to their higher profit margins., some healthy products could be (more) profitable (than unhealthy ones). [A]
Structuring Elements [Structure]	Organisational structures	Management structure<br>• Organisation is a cooperative, and has no shareholders to report to.<br>• Organisational and private stores depend on the organisation for essential services (e.g. replenishment, space planning, marketing campaigns).<br>• Managers of organisational stores are appointed by the organisation and can change regularly; private stores are managed by the owner, who have substantial freedom in their choices.<br>• The management of products (offer, prices, identity, etc.) is done by 'product managers', each responsible for select groups of products (e.g. 'fruits, potatoes, and vegetables').<br>• Shelf-space planning is overseen by 'space managers'.	- Support of store owners is essential for implementation in their stores, they have the power to stop components whenever they see fit. [1]<br>- Management of organisational stores can switch during implementation, which can make building relations difficult. [2a]<br>+ Initial acceptance of more radical ideas can be facilitated by support from influential or authoritative actors (e.g. upper management). {1/2]
		Performance metrics<br>• Management at product and store levels is judged through performance metrics.<br>• For store management: turnover, product waste, and salary cost.<br>• For product management: turnover, product waste, and profit margins.	- Current metrics push commercial efficiency over health. [A] Actors were unsure how to incorporate health more. [2a/3]<br>- Store management was concerned about waste and salary costs [2b], product management about waste and profit margins [1/2a].<br>+ There was some willingness to exclude pilot stores from these metrics. [2a]
		Marketing policies<br>• A 'formula' guideline is used to maintain coherence between stores and departments in terms of look, feel, and identity. e.g. communication style, placement, themes.<br>• The formula was recently revised to a more minimalistic style, which is regarded to improve customer experience. The changes are widely supported, but not yet fully integrated.	• Actors at both central and store level considered it of high importance that intervention components fit within the formula guidelines. [A]

		Resources<br>• Organisational resources are vital for any practices, including implementation of intervention components.<br>• Relevant resources are: floor space, shelf space, man-hours, expertise for specialised tasks, knowledge regarding health, money.<br>• The allotted use of various resources (time, people, money, space) is dictated by central policies. Privately-owned stores are freer in this regard.	- Human resources for intervention execution (expertise, man-hours) were often perceived as limited. [A]<br>- Actors perceived the organisation to lack the necessary authority and knowledge to make well-supported health claims. [1/2b/3]<br>- Staff motivation for component implementation at stores was at times perceived as low. [2/3]<br>- Specific presentation spaces (baskets and end-of-aisle shelves) are limited and occupied by other activities for some stores. [2/3]
	Physical structures<br>• Stores plan floor and shelf use around limited space, which is optimised and planned out in floor-plans and planograms. These vary between stores due to wide variations in store surface.<br>• The shelves/presentation space at various spots in the store are designed for specific products/product groups (e.g. a cooling, or specific size and shape).<br>• The shopping carts/baskets vary between stores. Some designs do not suit themselves to communication messages (no space, badly visible bottom), whereas others do.		- Floor and presentation space in stores is often limited and in high demand. [2/3]<br>- The shelves at check-outs only accommodate specifically shaped products (e.g. candy-bars, gum) [2b]<br>- In some stores the shopping baskets and carts are unsuitable to carry communication messages. [3]<br>+ Several methods/tools exist to create/adapt spaces for product presentation or signage (movable coolers, shelf-strips, extra baskets). [2/3]
	Organisational systems<br>• These are set up to assist the stores in their practices and alleviate workload.<br>• Deviations are possible, but labour intensive to maintain.	Planograms<br>• These plans specify the placement of products on store shelves, with each shelf having its own plan. Variations dependent on the size of the store are available.<br>• The plans incorporate sales data, information from market studies, and aim to maximize turnover and limit restocking work.<br>• They are developed and maintained centrally, by the space managers.<br>• The plans receive several updates per year, which are send out to the stores.	- Components deviating strongly from the existing planograms requires a new plan to be developed and maintained (centrally) or continuous maintenance (locally). Both are options labour intensive for either central or store management. [A]
		Supply and stocking systems<br>• The replenishment of stores is automated. The system links directly to planograms, and uses this information to predict orders. Deviations from the planogram require manual adjustments to these orders.	- Deviations from planograms have repercussions for the replenishment of products, which then need to be directly overseen by store management. [A]
		Price systems<br>• Product prices are managed through a central system, which does not allow deviations. Price changes in specific stores thus need to be maintained locally.<br>• The local systems have options for temporary price decreases, but not increases.<br>• A complete restructuring of the pricing system is being planned, which may bring new options.	- A new price-system will be implemented across all stores. The exact capabilities of the system are yet unknown, and will likely present new/unique barriers to any price related intervention components. [2a/3]<br>- The centralized nature of the current price system means that local price increases are unfeasible. [2a/3]
		Campaigns & activities	+ This channel can be utilised to communicate intervention plans to stores in an established way. [A]

		• Stores organise various campaigns and activities. These are communicated by the central office as scripts, to be carried out as written.	
	Products	**Qualities**<br>• Various inherent qualities of products limit what intervention approaches are possible, e.g. shelf-life, shape, ease of preparation, healthiness.	- The use of products for certain promotion methods differs based on qualities, e.g. ease of preparation, function in diet, taste, healthiness. [2/3]<br>- Products can have various requirements for their presentation (temperature, shape) in the store, limiting options to place items together. [2/3]
		**Range**<br>• Range of products differs per group. Some have a high variety, spread in sales, or varying shelf requirements, others low variety, or a few dominant products.	- Range characteristics limit what can be done with components involving relative pricing or positioning within a group. [2/3]<br>+ Various product groups were perceived to have comparable healthy and unhealthy alternatives, between which switching could be promoted, or healthy options to be promoted in general. [2b/3]
		**Price & profitability**<br>• The profitability of products depends on volume sold and profit margin. Most sales volume is cheaper, highly processed, often unhealthy products, with relatively low margins. Fresh produce often has bigger margins, due to lower overhead costs, but also lower sales volume. Both types are potentially profitable.	- Some actors perceived unhealthy products as more profitable due to the perceived higher interest in them. [A]<br>- Some actors regarded healthy products as less profitable, due to the perceived low interest in them, and lower margins. [2b/3]<br>- The profit margins products become smaller when their prices are reduced. This places a limit on how large price reductions on healthy products can be. [A]<br>+ Some actors perceived healthy (fresh) products as more profitable, due to higher margins. [2/3]
Practices	**Marketing practices**<br>• There are multiple practices aimed at promoting products:<br>○ Second placement, increased visibility, increased product facings, price promotions and their communication, folder compositions, and tastings.<br>• Practices vary between stores, due to the ongoing implementation of the revised formula. Parts of the old style are regarded as redundant or ineffective. (e.g. floor/ceiling communication, high number of shelf signs), whereas new style and retained practices are regarded as effective and worth their cost.		- Similarity to ineffective {A} or redundant [2b/3] practices leads to lower trust and perceived worth of a component, or higher perceived costs/effort.<br>+ Similarity to successful or promising practices leads to higher trust and perceived worth of a component, or lower perceived costs/effort.
	**Health promotion practices**<br>• The organisation engages in several health promotion practices outside the intervention:<br>○ Sugar content labelling in sugar-sweetened beverages, healthy choice tags, vegetable intake recommendations, healthy recipes, and salt reduction in bread.		- These practices create familiarity with and acceptance for health promotion and its potential approaches for organisational actors. [A]
	**Side projects**<br>• Practices outside the core business of running stores:<br>○ Acquired new stores and has been working to get these ready for operation, working to implement a new price/check-out system.		- These activities have placed strain on organisational resources. [A]<br>- The introduction of the new price/check-out system limits what is possible with price changes in stores which need to change during the intervention. [2a/3]
Actors	**Personal preferences**<br>• Throughout the process, key-actors expressed personal likes or dislikes for certain components. Based on either intuition or comparisons to previous experiences.		• These preferences seemed to occasionally influence actors' dispositions towards specific components. [A]
	**Engagement**		- Some components were made negotiable by the support of influential actors at the central office. [2a]

	• Through the co-creative process, we sought to engage key-actors throughout the organisation to build support for the intervention.		+ Directly engaging store staff was perceived to increase their commitment by the store managers [2b].
External Influences<br>*(note: these represent perceptions from supermarket actors of these external factors)*	Market	Competition over customers<br>• The various supermarket chains compete heavily over consumers. Poaching the customers of other chains is common, particularly based on pricing.	- Actors perceived risk of losing customers to other chains due to frustration or annoyance with intervention components. [1/2b/3]<br>+ One Store had little direct competition, lessening the risk of losing customers. [3]
		Price comparisons<br>• The media often hold price comparisons between stores, which are regarded by the industry as a major influence on where consumers go as customers.	- Actors feared that comparisons in intervention stores could negatively affect the consumers' price-perception in the country, losing them customers. [A]<br>+ Chances for a pilot store to be used for a price comparison were perceived to be quite low. [2a/3]<br>+ The goal of the price increases, and the combination with price decreases, was perceived to have a morally strong message, which could be given a positive spin. [3]
		Health-focussed trends<br>• Various supermarkets recently engaged in health-focussed marketing strategies, meant to leverage a perceived increase in consumer interest in health.	+ Promoting health seems to be a growing strategy for supermarkets, which the organisation wants to invest in as well. [2/3]
	Industry & Intermediaries<br>• Relations with the industry are important for product managers. Industry can lobby for preferential treatment of their products in terms of promotion or placement in a store, e.g. by subsidising price promotions or folders. They can also provide information from market studies for the management of product groups.<br>• Most negotiations with the food industry are performed through a buyers' club. These consist of several supermarket chains together. Not all chains have the same priorities with their products, and a compromise needs to be reached in what products are carried. All members are required to carry the same product out of negotiated options.		- Some components would treat products from specific brands less favourably, potentially harming relations with those industry players. [2b]<br>- Stores are required to carry products negotiated by the buyers' club. Therefore, dropping such products is not an option. [1]
	Customers<br>• Organisational customers are generally of middle and higher age, and relatively traditional (from a Dutch perspective) and habitual in their shopping behaviour.<br>• The customers living in more remote areas, around potential pilot stores for the intervention, are generally of lower socioeconomic status.<br>• The customers are perceived to, on average, have lower knowledge and skills regarding healthy diets, food preparation, and interpreting food labels and symbols.<br>• The perceived interest of customer in the topic of health varied between areas.		- Components, especially involving symbols and product information were perceived as possibly too complex for customers to understand. [A]<br>- Various components were perceived to not take specific characteristics of customers into account (e.g. preferences, interests, financial means, knowledge, shopping habits). [A]<br>- In certain areas theft/vandalism by customers was perceived as a substantial issue. [2/3]<br>- Interest in health is still perceived to be low, especially in more rural areas. [2/3]<br>+ Growing interest in health is observed in consumers. [2/3]<br>+ Various components were perceived to closely fit specific characteristics of customers into account (e.g. preferences, interests, financial means, knowledge, shopping habits). [A]
	Legislation<br>• Governmental policies guide/limit some practices in supermarkets, e.g. product composition, health claims, and food-safety precautions		- Legislation limits what claims can be made on products with regards to effects on health. [1]

		- Health/hygiene legislation can lead to extra workload for stores from tastings, especially for fresh products which have more strict guidelines. [3]
